# Changes in the Structure of the Microbial Community Associated with *Nannochloropsis salina* following Treatments with Antibiotics and Bioactive Compounds

**DOI:** 10.3389/fmicb.2016.01155

**Published:** 2016-07-26

**Authors:** Haifeng Geng, Mary B. Tran-Gyamfi, Todd W. Lane, Kenneth L. Sale, Eizadora T. Yu

**Affiliations:** ^1^Department of Systems Biology, Sandia National LaboratoriesLivermore, CA, USA; ^2^Department of Biomass Science and Conversion Technology, Sandia National LaboratoriesLivermore, CA, USA; ^3^Institute of Chemistry, University of the Philippines DilimanQuezon City, Philippines

**Keywords:** correlation network, microbiota, algae

## Abstract

Open microalgae cultures host a myriad of bacteria, creating a complex system of interacting species that influence algal growth and health. Many algal microbiota studies have been conducted to determine the relative importance of bacterial taxa to algal culture health and physiological states, but these studies have not characterized the interspecies relationships in the microbial communities. We subjected *Nanochroloropsis salina* cultures to multiple chemical treatments (antibiotics and quorum sensing compounds) and obtained dense time-series data on changes to the microbial community using 16S gene amplicon metagenomic sequencing (21,029,577 reads for 23 samples) to measure microbial taxa-taxa abundance correlations. Short-term treatment with antibiotics resulted in substantially larger shifts in the microbiota structure compared to changes observed following treatment with signaling compounds and glucose. We also calculated operational taxonomic unit (OTU) associations and generated OTU correlation networks to provide an overview of possible bacterial OTU interactions. This analysis identified five major cohesive modules of microbiota with similar co-abundance profiles across different chemical treatments. The Eigengenes of OTU modules were examined for correlation with different external treatment factors. This correlation-based analysis revealed that culture age (time) and treatment types have primary effects on forming network modules and shaping the community structure. Additional network analysis detected *Alteromonadeles* and *Alphaproteobacteria* as having the highest centrality, suggesting these species are “keystone” OTUs in the microbial community. Furthermore, we illustrated that the chemical tropodithietic acid, which is secreted by several species in the *Alphaproteobacteria* taxon, is able to drastically change the structure of the microbiota within 3 h. Taken together, these results provide valuable insights into the structure of the microbiota associated with *N. salina* cultures and how these structures change in response to chemical perturbations.

## Introduction

Open algal cultures are complex dynamic ecosystems inhabited by diverse microbial communities (Carney et al., 2014). In many cases, microbes in microalgae ecosystems have effects on algal physiology and nutrition as well as play critical roles on stability and homeostasis of algal ecosystems in both lab cultures and field studies (Kayser, 1979; Lee et al., 2000; Geng and Belas, 2010b). These types of algal-microbial interactions have been shown to either promote algal growth (Hold et al., 2001) and protect algae from invading pathogens (Geng and Belas, 2010b), or to inhibit algal growth and destabilize the algal ecosystem (Cole, 1982; Carney et al., 2014). Thus, knowledge of the composition and structure of algal-associated microbiota is important for understanding homeostasis in robust algal cultures, as aberrant microbiota have been reportedly linked with precipitous crashes of algal cultures (Cole, 1982; Carney et al., 2014). These observations argue for finding important relevant factors that contribute to the appropriate composition and proper structuring of this complex biological community (Carney et al., 2014; Sison-Mangus et al., 2014).

Given that microbial communities are enormously diverse, we are only able to characterize their diversity with the recent use of next-generation sequencing technologies (Gonzalez et al., 2012). In the past, algal microbiota researchers have focused on understanding the relationship between collective population diversity and environmental conditions in algal cultures (Alavi et al., 2001; Carney et al., 2014) and on linking individual membership composition to ecosystem descriptors (Costello et al., 2009). However, the overarching property of a microbial consortium may stem from the requisite biological functions of collective groups consisting of multiple interacting bacterial species (known as modules) found in the community. For example, biofilm formation and metabolic complementation are modules in which the collective bacteria species deliver the required functions (Raes and Bork, 2008; Geng and Belas, 2010b). However, limited information is available on the substructure of the microbial communities (e.g., formation of bacterial groups or modularities recapitulated from interacting bacterial species) associated with microalgae. This not only hinders the interpretation of topological structures that oversee the proper function of the microbial community but also reduces the reliability of prediction of the community structure and its effects on the long-term stability of microalgae cultures.

One way of exploring substructures in biological systems is to look for pairs of entity relationships and subsequently using this information to build a correlation network of potentially interacting entities. Correlation networks have been successfully used in studies of cancer (Choi et al., 2005), yeast genetics (Ge et al., 2001), and microbial ecology (Lovejoy et al., 1998; Duran-Pinedo et al., 2011; Gilbert et al., 2012), which focused on searching for groups of lineages that occur together more often than expected by chance. Once networks have been built, several measures and metrics such as node centrality and betweenness can be evaluated to assess and identify the most important and influential nodes in the network (Jeong et al., 2001). Centrality measures the importance of a node based on the number of connections it makes with other nodes (degree centrality) in the network and may include measures of the importance of the neighbors to which it is connected (eigenvalue centrality, Katz centrality, page rank). Nodes with high number of connections to other nodes are perceived as having greater influence over the entire network (Jeong et al., 2001). Betweenness centrality measures the extent to which a node lies on paths between other nodes and is an indicator of the influence a node exerts over other nodes in the network. Nodes with higher betweenness are perceived to have greater influence within a network by virtue of their control over information passing among the other nodes in the network (Yoon et al., 2006). In other microbial ecological systems, network analyses based on Weighted Correlation Network Analysis (WGCNA) were used to identify hub OTUs with influential roles in maintaining a mature biofilm (Duran-Pinedo et al., 2011).

Recently, we found a microbial community associated with *Nannochloropsis salina* (CCMP, 1776) that displayed community stability and resilience to environmental perturbations at a global level (Geng et al., 2016). To obtain clues about the substructuring of the microbial community associated with *N. salina*, we subjected *N. salina* containing communities to various chemicals (e.g., antibiotics, metabolites) and examined changes in the connectivities among taxa in the microbial community. We used longitudinal 16S gene amplicon sequencing of 23 samples descended from a single ancestral microbiota and built correlation networks to reflect the dynamics of the inter-taxa associations and to investigate variations in taxa organization in response to different chemical treatments. As a result, we identified five cohesive modules representing various chemical treatment responses. Subsequently, through network centrality analysis, our data showed that key nodes in the modeled network were primarily from the bacterial species belonging to *Alteromonadeles* and *Alphaproteobacteria*, suggesting species from these groups are of particular significance and serve as “keystone” OTUs in the microbial community associate with *N. salina*.

Species of the *Roseobacter* clade of *Alphaproteobacteria* are important symbionts of microalgae (Gonzalez and Moran, 1997; Treangen et al., 2013). One of the key aspects of the *Roseobacter*–microalgae symbiosis is founded on *Roseobacter*'s ability to produce a distinct set of infochemicals or signaling compounds with specialized functions (Mayali and Azam, 2004; Geng and Belas, 2010b; Seyedsayamdost et al., 2011; Treangen et al., 2013). For example, the antibiotic tropodithietic acid (TDA) produced by *Roseobacter* played a pivotal role in the bacterial-algal symbiosis by regulating TDA gene expression across various species (Geng and Belas, 2010a; Porsby et al., 2011) and preventing bacterial infection during prosperous algal blooms (Brinkhoff et al., 2004; Bruhn et al., 2007). Inspired by insights from network analysis, we treated the microbiota with various concentrations of TDA, simulating TDA secretion from some *Alphaproteobacteria*. The introduction of TDA drastically impacted community structure at a global level. Taken together, this experimental metagenomics study, while not fully characterizing OTU interactions in the microbial community associated with *N. salina*, provides a valuable framework to aid modeling of interactions among the algal microbiota and understanding the important microbiota—microalgae interactions in the context of the complexity of the studied ecosystem.

## Materials and methods

### Algal cultures and experimental design

Artificial microcosms of *N. salina* (CCMP, 1776) and the microbiota were generated by acclimating *N. salina* culture together with seawater microbiota from the coast of Santa Cruz, CA (Geng et al., 2016). After 1:10 culture dilution with fresh sterile artificial seawater media (ESAW) (Berges et al., 2001), algal cultures were grown to exponential phase. For the community restructuring experiments, triplicates were generated by splitting the algal cultures into 8 ml/well in 6-well Corning Costar® cell culture plates. The aliquoted exponential phase *N. salina* cultures (day 4 post-inoculation) were immediately spiked with two separate doses of sterile-filtered organic compounds (dosage were chosen based on the typical working concentrations for each chemical and a reference concentration (500 nM) for all chemicals; Table 1): (i) mixtures of common forms of bacterial quorum-sensing signaling molecules (Atkinson and Williams, 2009), including acyl-homoserine lactones (AHLs) [Sigma Aldrich (St. Louis, MO)] composed of N-butyryl-DL-homoserine lactone, N-hexanoyl-DL-homoserine lactone, N-octanoyl-DL-homoserine lactone, N-(β-Ketocaproyl)-L-homoserine lactone [31 nM (Wagner-Dobler et al., 2005) and 500 nM]; (ii) tetracycline (500 nM and 104 μM; Sambrook et al., 1989); (iii) ampicillin (500 nM and 134 μM; Sambrook et al., 1989); (iv) tropodithietic acid (TDA), an antibacterial and chemical signaling compound (31 nM and 500 nM; D'Alvise et al., 2012; Enzo Scientific, Farmingdale, NY); (v) D-glucose (500 nM and 300 μM) [Sigma Aldrich (St. Louis, MO)]. Low doses of D-glucose (500 nM) were treated as negative controls, as the effect of glucose on the bacterial community has been found to be minimal compared to antibiotic treatment (Dandekar et al., 2012). The incubation continued at 21°C under constant light conditions (100 μmol photons m^−2^s^−1^). An initial 4 ml was taken from each sample after 3 h and centrifuged 10,000 g for 5 min to pellet the bacterial community. The remaining 4 ml in each well were harvested after 24 h. Bacterial community pellets were stored at −80°C prior to DNA extraction.

**Table 1:** **Chemical treatments in algal microcosms**.

**Sample name**	**Chemicals^a^**	**Treatment time (hours)**	**Number of sequences**	**Observed OTUs^b^**
starth0	Blank	0	81,967	3731
AHLL3	AHLs (31 nM)	3	114,404	3711
AMPL3	Ampicillin (500 nM)	3	103,175	3774
GLUCOSEL3	Glucose (500 nm)	3	159,219	3550
TDAL3	TDA (31 nm)	3	121,794	3698
TETL3	Tetracycline (500 nm)	3	138,805	3615
AHLH3	AHLs (500 nm)	3	114,275	4029
AMPH3	Ampicillin (134 μM)	3	121,688	4208
BLANK3	Blank	3	110,341	3759
GLUCOSEH3	Glucose (300 μM)	3	137,209	3778
TDAH3	TDA (500 nm)	3	122,655	4176
TETH3	Tetracycline (104 μM)	3	146,511	4104
AHLL24	AHLs (31 nm)	24	223,733	3036
AMPL24	Ampicillin (500 nm)	24	135,886	3857
GLUCOSEL24	Glucose (500 nm)	24	138,265	3360
TDAL24	TDA (31 nm)	24	245,234	3320
TETL24	Tetracycline (500 nm)	24	242,666	4100
AHLH24	AHLs (500 nm)	24	180,657	3606
AMPH24	Ampicillin (134 μM)	24	130,815	3732
BLANK24	Blank	24	128,023	3704
GLUCOSEH24	Glucose (300 μM)	24	242,402	2892
TDAH24	TDA (500 nm)	24	203,911	4130
TETH24	Tet (104 μM)	24	311,800	2875

a *Chemical name (final concentration)*.

b *Observed OTUs/sample rarefied at 80,000 sequences per sample*.

### Samples and 16S rRNA gene sequencing

Genomic DNA was extracted from algal culture samples with associated microbiota using a ZR Fungal/Bacterial DNA MiniPrep (ZYMO Research, Irvine, CA) following the manufacturer's protocol. 16S gene PCR preparation with standard procedures with barcoded primer set 341F forward and 518R reverse primer as previously described (Bartram et al., 2011). The triplicate PCR products from each sample were pooled and purified using QIAquick PCR purification kit (Qiagen, Valencia, CA) followed by quantification on a Nanodrop ND spectrophotometer (Thermo Science, Wilmington, DE). Twenty-three samples in equal amount with unique index sequence were mixed and further subjected to 2% gel purification using QIAquick gel extraction kit (Qiagen) following by quantification with a Bioanalyser DNA 7500 chip (Agilent Technologies, Santa Clara, CA). The prepared 16S rRNA gene library with addition of 30% PhiX was sequenced for 151-nucleotide paired-end multiplex sequence on MiSeq (Illumina, Hayward, CA) with a loading concentration of 8 pM following manufacturer's protocol.

### Sequence processing

Sequence reads were filtered to remove sequences of poor quality (e.g., two or more continuous base calls below 30 and length <75 bases). Forward and reverse sequences of paired-end sequences were assembled by aligning 3′ ends using SHE-RA software (Rodrigue et al., 2010). Stitched sequences were then clustered using the UCLUST algorithm with 97% similarity and assigned to operational taxonomic units (OTUs) above a 0.80 confidence threshold, which is a homology cutoff value above which typically denotes bacterial species, and taxonomic classifications were assigned in reference to Greengenes taxonomy (RDP-Classifier) in QIIME (DeSantis et al., 2006; Caporaso et al., 2010; Sul et al., 2011). OTUs ascribed to chloroplasts were then excluded from the pre-trimmed OTU table. Differences between samples (beta diversities) were performed using QIIME rarefied at depth 80,000 sequences/sample in post-trimmed OTUs table (DeSantis et al., 2006; Caporaso et al., 2010). To obtain relative abundances of OTUs per sample, the post-trimmed OTU reads were divided by the sum of usable reads. Principal coordinate analysis (PCoA) was applied based on their between-samples weighted UniFrac distances metrics. Jackknifing was performed by resampling 1000 times with replacement of 50,000 sequences per sample in post-trimmed OTUs table and was used to build a rooted pairwise similarity tree using the Unweighted Pair Group Method with Arithmetic Mean (UPGMA) in QIIME (Caporaso et al., 2010)

### Correlation network analysis

To remove poorly represented OTUs and reduce network complexity, we filtered and retained OTUs that were observed in a minimum of 13 out of total 23 samples, resulting in 1766 OTUs out of 27,298 OTUs being selected. Based on this, we calculated all pairwise Pearson correlations between OTUs using the WGCNA module in R (Team, 2015). Rather than focusing on the significance of the correlation, soft thresholding power was applied in WGCNA to dynamically prune branches off the dendrogram depending on clusters shape (DiLeo et al., 2011). To minimize spurious associations during module identification, the adjacency matrix was transformed to a Topological Overlap Matrix, corresponding dissimilarity was then calculated as a robust measure of interconnectedness in a hierarchical cluster analysis (DiLeo et al., 2011). Average linkage hierarchical cluster analysis was used to construct the corresponding dendrogram (DiLeo et al., 2011).

### Network centralities

Networks were explored, analyzed and visualized with Cytoscape based on the pairwise correlations (Smoot et al., 2011). Global measurements (e.g., betweenness, closeness, neighborhood connectivity, and topological coefficient) assessing the topology and centrality of the resulting network were calculated using Cytoscape with the NetworkAnalyzer plugin (Doncheva et al., 2012). To relate modules to chemical treatments, we correlated the eigengene for each module with the chemicals and looked for significant associations based on *p*-values.

## Results

### Response of the microbial community associated with *N. salina* to chemical perturbations

**Figure 1:**
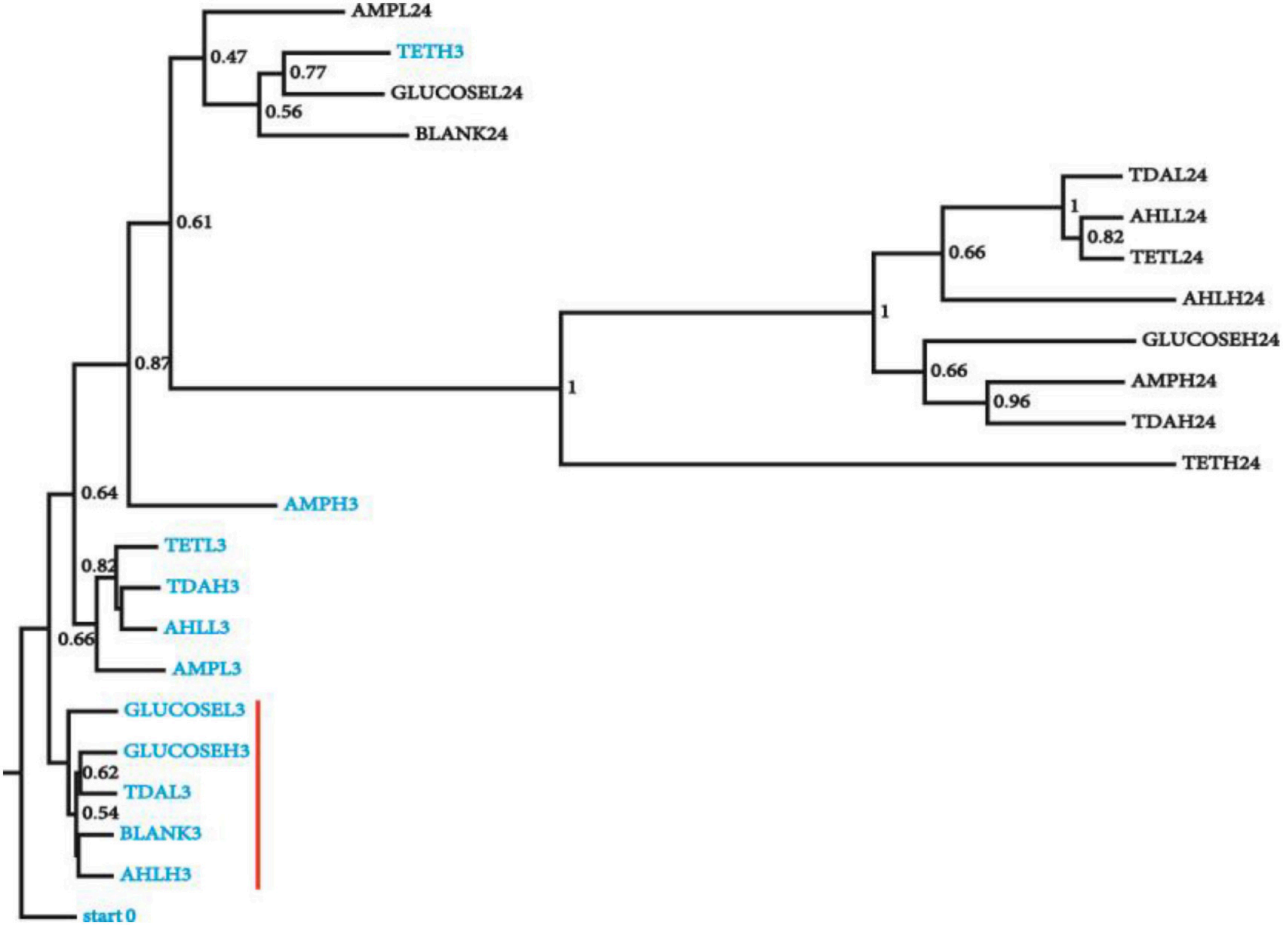
**Clustering of microbial diversity (β-diversity) of the starting microbiota with the samples from different chemical treatments at 3 and 24 h**. Jackknifing of the UPGMA tree displays the robustness of clustering of the microbiota from the 3 h from 24 h samples. Bootstrap values are shown at the nodes of the tree, indicating percentage of jackknifed data supporting the node. Samples are named as following: chemical name (e.g., GLUCOSE), H/L (high/low concentration, e.g., L) and treatment time (e.g., 3), sample (GLUCOSEL3, treated with low amount of glucose for 3 h). Samples from 3 h (blue) treatment that were most similar to the starting microbiota are highlighted with a red vertical red bar.

To generate a collection of datasets representing species relative abundance in response to known perturbations, we added a set of chemicals expected to have pervasive effects on *N. salina* microbial communities to the culture during exponential growth phase. The spiked chemicals include AHLs mixtures, tetracycline, ampicillin, TDA, and D-glucose in low and high concentrations chosen to maximize the likelihood of microbiota perturbations (Section Materials and Methods). The microbial community associated with *N. salina* from one untreated and four treated samples were collected at 3 and 24 h post inoculation, along with one starting microbiota (0 h), collectively generating 23 samples (Table 1). A total combined 21,029,577 reads from rRNA 16S gene V3 region of 23 microbiota samples were successfully assigned to individual samples. After filtering out low quality reads and OTU assignments (Section Materials and Methods), we obtained an OTU table of the 23 samples with a mean of 158,931 Seqs/sample (minimum: 81,976; maximum: 311,800; median 137,209 Seqs/sample) (Table 1). We assessed inter-sample variability using PCoA based on unweighted Unifrac metric measures in which the distance represents dissimilarity among community structures (Figure 1) (Lozupone and Knight, 2005). Among all tested microbiota, the initial microbiota (start0), blank control (3 h) and glucose treated samples (3 h) were found to be most similar in composition, thus clustering to what we call the “early” microbiota group (Figure 1, labeled with the red bar). The similarities among these groups reflect glucose and short temporal lags have only marginal effects on the structure of the microbiota. These patterns are expected, as glucose is widely used by many bacteria through the hexose monophosphate pathway in glucose metabolism, thereby having minimal effects in restructuring the microbial assemblage as compared to antibiotics (Dandekar et al., 2012). In contrast, short-term 3-h low concentration antibiotics treated-samples (TETL3 and AMPL3) migrate further away from the starting sample (start0) (Figure 1). This shift is further pronounced when comparing the starting microbiota and the high-dose antibiotics-treated samples (TETH3 and AMPH3) at the same 3-h timepoint (Figure 1). In addition, the short-term low TDA treatment for 3 h (TDAL3) resulted in a microbiota that coincided with “early” group microbiotas, which are most similar to the starting microbiota, whereas the high TDA imposed pronounced effects on the microbiota.

The microbiotas from the 3 h time points clustered together, while the 24 h treatment microbiotas formed a separate cluster (Figure 1), revealing groupings in the bacterial community as a function of treatment time. Collectively, 16S gene profiles displayed both culture age-dependent and chemical-responsive structural rearrangement of algal microbiota.

### Structural modulation of algal microbiota upon chemical treatments

**Figure 2:**
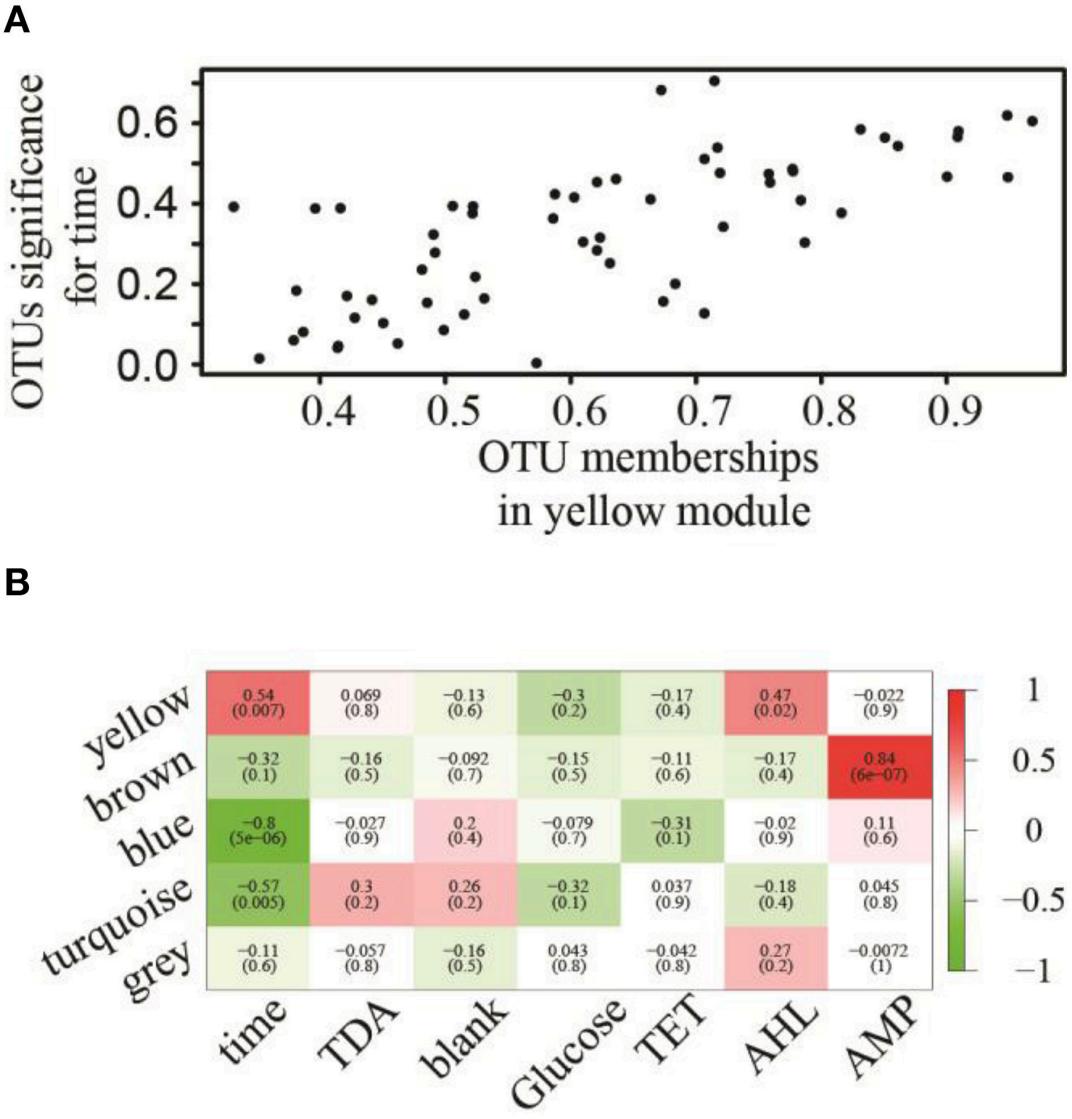
**Association of modules with external treatment factors. (A)** OTUs associated with treatment–time with respect to their membership significance belonging to the yellow module. **(B)** Eigengene adjacencies heatmap identifying modules (row) that significantly associated with chemical treatments (column). Negative correlations (green) and positive correlations (red) indicate high adjacency (DiLeo et al., 2011), while white (zero) indicates low adjacency.

**Figure 3:**
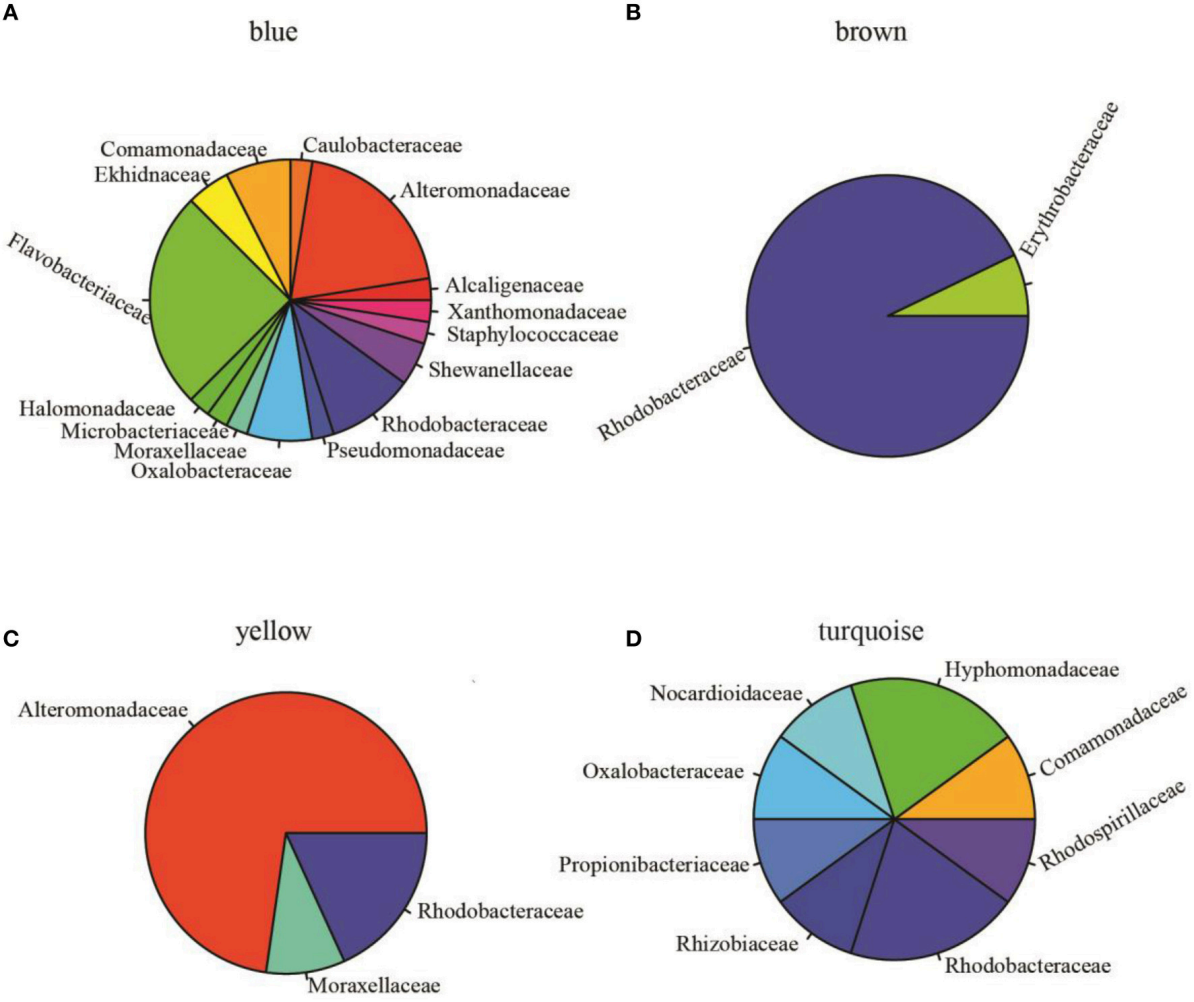
**Relative abundance of OTUs at the family level (indicated by different colors) in identified modules. (A)** Blue module (total OTUs 40 annotated at family level, 127 OTUs unassigned at family level are not shown) was dominated by *Flavobacteriaceae* (25%, *n* = 10) and *Alteromonadaceae* (20%, *n* = 8). **(B)** Brown module (14 OTUs annotated, 63 unassigned) was dominated by *Rhodobacteraceae* (92%, *n* = 13). **(C)** Yellow module (total OTUs 11 annotated at family level) is comprised of *Alteromonadaceae* (72%, *n* = 8), *Rhodobacteraceae* (9.1%, *n* = 1), and *Moraxellaceae* (9.1%, *n* = 1). **(D)** Turquoise module (10 OTUs assigned to 8 families, 876 unassigned OTUs) is comprised of 8 families. These data showed that *Rhodobacteraceae* was significantly enriched in the brown module (one-sided Fisher's exact test, *P* < 0.0001) and *Alteromonadaceae* was significantly enriched in the yellow module (one-sided Fisher's exact test, *P* < 0.05).

To assess pairwise Pearson correlations among OTU relative-abundance data, we randomly selected 80,000 sequences per sample, built OTU tables, and filtered OTUs that contributed to at least half of 23 microbiota samples. The 1766 qualified OTUs produced from this process were subsequently fitted to a correlation matrix with scale free topology (power of 6, *R*^2^ = 0.81) using WGCNA, which has been shown to be robust for analyzing relative abundance data (DiLeo et al., 2011). WGCNA revealed five major co-abundance modules that were arbitrarily given colors yellow, brown, blue, turquoise, and gray (Figure S1). Upon identification of modules in the microbial community associated with *N. salina*, we associated them with external treatment factors. Figure 2A shows that OTU membership in the yellow module was positively correlated with culture-age associated OTUs (coefficient = 0.54, *P* < 0.05). Figure 2B illustrates that treatment time was especially important, as three modules were found to be significantly associated with treatment time, whereas the blue (coefficient = −0.80, *P* < 0.05) and turquoise (coefficient = −0.57, *P* < 0.05) modules were negatively correlated with treatment time. These data indicate treatment time was a primary factor in contributing to variations in species abundance in these particular studies, which is consistent with beta-diversity analysis using unweighted Unifrac metric measure (Figure 1). Neither the glucose treatment nor the untreated microbiota was significantly associated with any of the five identified modules (*P* > 0.05). There were also no modules significantly associated with tetracycline under the studied concentrations. Figure 2B shows the brown colored module was selectively correlated with ampicillin treatment (*P* < 10^−7^). Taxonomy analysis showed the brown chemotype-specific module was enriched by the *Rhodobacteraceae* family (13 out of 14, with only 1 coming from the *Erythrobacteraceae* family, *P* < 0.05, enrichment analysis), whereas the yellow module was enriched by the *Alteromonadaceae* family (8 out of 11, *P* < 0.05, enrichment analysis) (Figure 3). Therefore, the apparent module substructures were associated with the taxonomical coherence of dominant OTUs.

### Network centralities and identification of hubs

**Figure 4:**
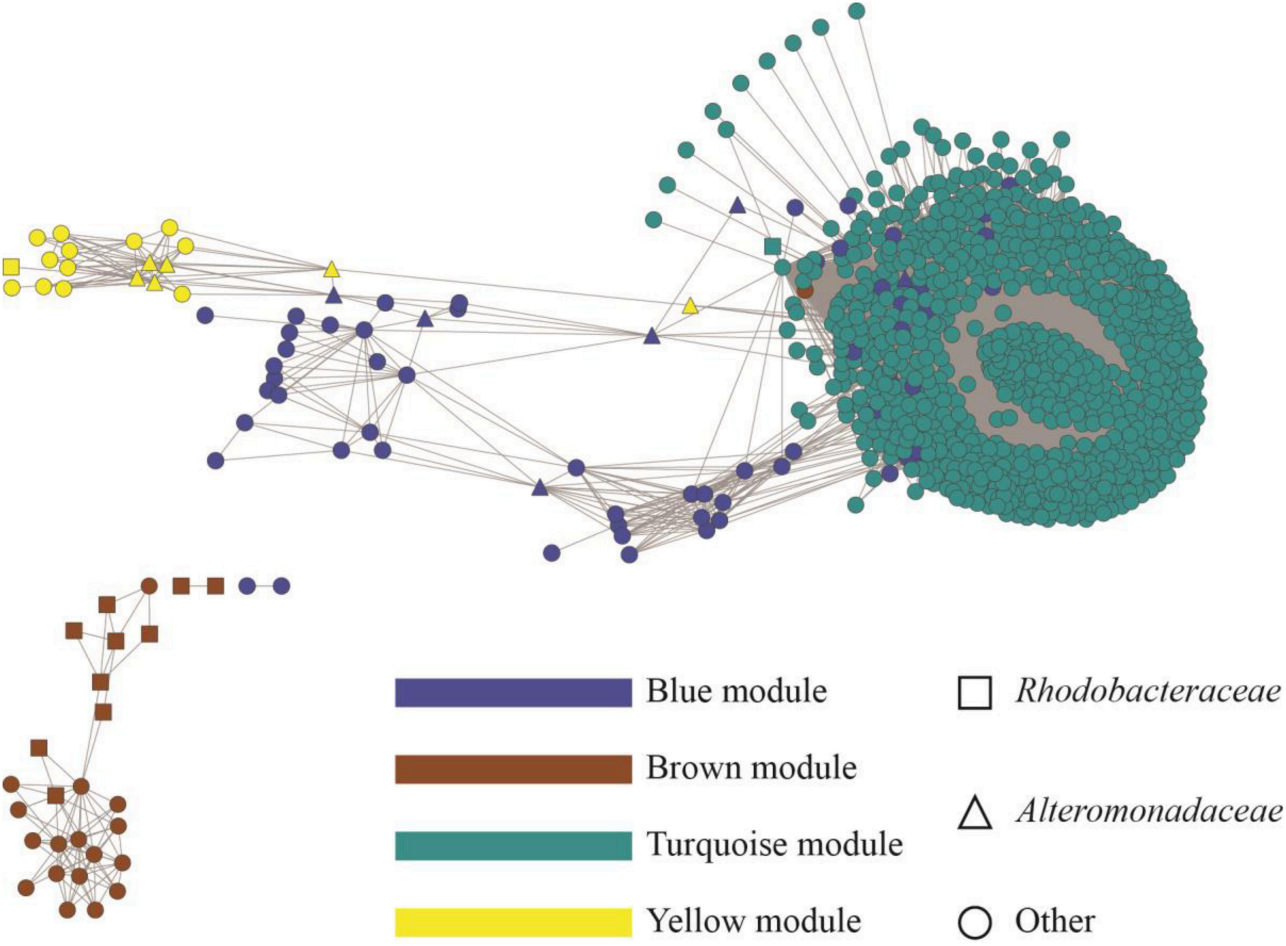
**Topology of the microbial community associated with *N. salina***. The identified modules were colored according to module names (Blue, Brown, Turquoise, and Yellow). The whole network contains 1000 nodes representing bacterial OTUs and 68,675 edges, showing correlations between the OTUs with an edge weight cutoff of 0.20 in the WGCNA network (DiLeo et al., 2011). OTUs were annotated at the family level. *Rhodobacteraceae* and *Alteromonadaceae* are shown as squares and triangles, respectively. OTUs from families other than *Rhodobacteraceae* and *Alteromonadaceae* are displayed as circle.

To display an empirical base for discovery and to explore specific details of the substructures of the community, associations from the top 1000 OTUs ranked by coefficient were plotted. Figure 4 shows the resulting microbial network consisting of 1000 nodes and 68,675 edges with average clustering network coefficient of 0.856, suggesting the down-selected OTUs constituted a highly connected network. The average number of network neighbors between all pairs of nodes was 137, and the network centralization was 0.768 as determined using NetworkAnalyzer (Doncheva et al., 2012). Hub nodes within respective modules are often proposed to be critical components for the network (Mayali and Azam, 2004). To identify species that may be acting as hubs, and thus be critically important components of the community network structure, we ranked nodes using various network centrality measures, including betweenness, closeness, neighborhood connectivity and topological coefficient (Table 2). Based on these measures, species from the *Alteromonas* and *Rhodobacteraceae* families were hypothesized to be key species influencing the inter-OTU relationships.

**Table 2:** **Identified “keystone” OTUs in co-abundance networks**.

**OTU ID^a^**	**Family^b^**	**Betweenness**	**Closeness**	**Neighborhood Connectivity**	**Topological Coefficient**	**Module Membership**
268	NA^c^	0.51	0.71	8	0.36	Brown
**179^d^**	***Rhodobacteraceae***	**0.29**	**0.51**	**5**	**0.28**	**Brown**
1070	NA	0.14	0.9	151	0.16	Turquoise
264	*Erythrobacteraceae*	0.11	0.63	8	0.41	Brown
265	NA	0.08	0.62	8	0.43	Brown
267	NA	0.07	0.6	9	0.45	Brown
1057	NA	0.06	0.85	161	0.17	Turquoise
**176**	***Rhodobacteraceae***	**0.05**	**0.38**	**3**	**0.47**	**Brown**
**91**	***Alteromonadaceae***	**0.05**	**0.49**	**276**	**0.3**	**Blue**
**126**	***Rhodobacteraceae***	**0.05**	**0.47**	**10**	**0.53**	**Brown**
1040	NA	0.04	0.82	165	0.18	Turquoise
1009	NA	0.03	0.81	163	0.18	Turquoise
1066	NA	0.03	0.8	165	0.18	Turquoise
266	NA	0.03	0.56	10	0.51	Brown
1087	NA	0.02	0.79	168	0.18	Turquoise
1048	NA	0.02	0.8	167	0.18	Turquoise
**131**	***Rhodobacteraceae***	**0.02**	**0.52**	**10**	**0.5**	**Brown**
257	NA	0.02	0.55	10	0.54	Brown
**89**	***Alteromonadaceae***	**0.02**	**0.48**	**643**	**0.7**	**Yellow**
**92**	***Alteromonadaceae***	**0.02**	**0.33**	**11**	**0.44**	**Yellow**

a *Assigned OTU identity*.

b *OTU taxonomy at family level*.

c *NA, OTU taxonomic assignment not available at family level*.

d *Proposed “keystone” OTUs Rhodobacteraceae and Alteromonadaceae are highlighted in bold*.

### TDA alter the structure of the microbial community associated with *N. salina*

**Figure 5:**
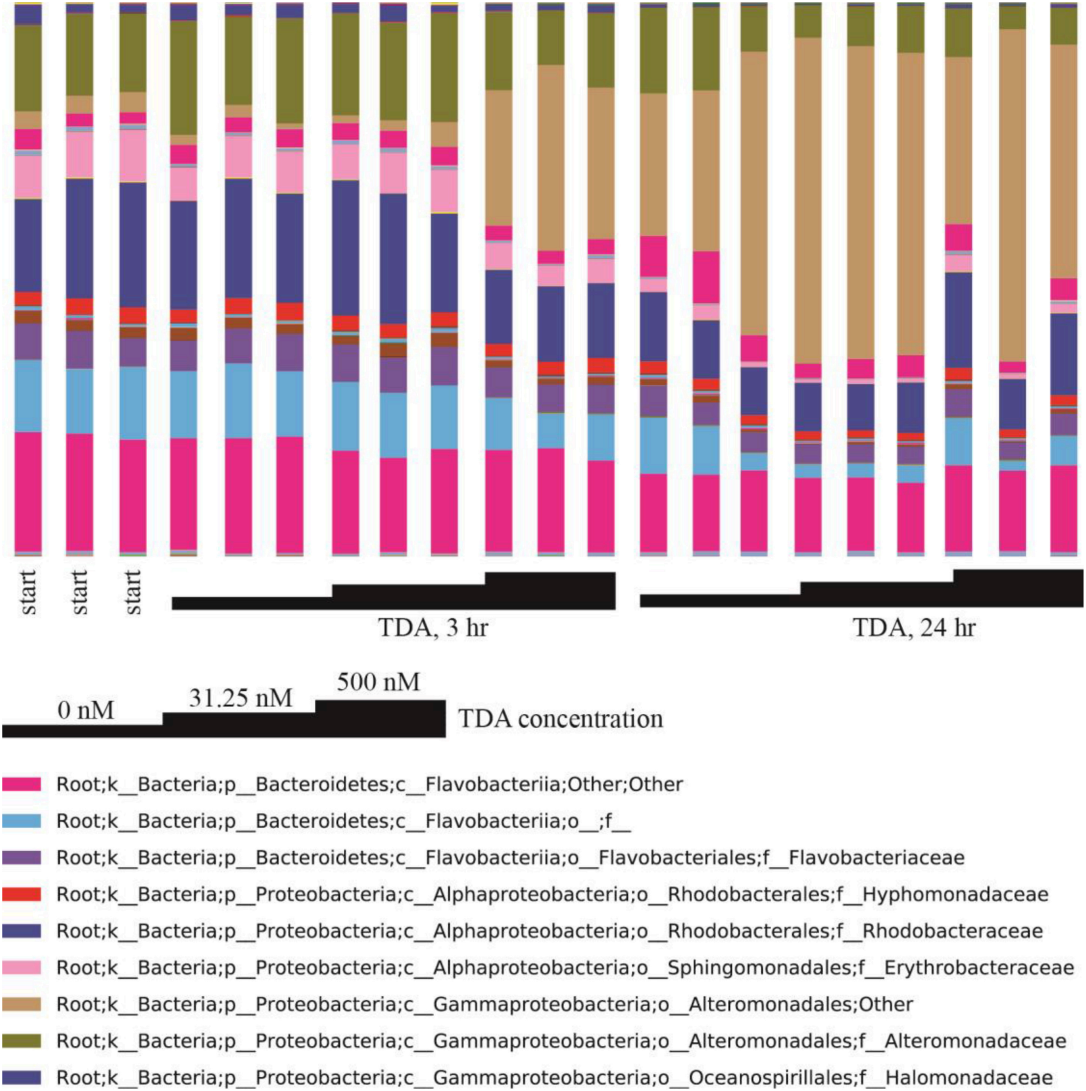
**Family-level taxonomic composition of TDA-perturbed microbiota**. Triplicate samples are grouped by TDA concentration and exposure time. Taxonomic affiliations of OTUs are shown at the family level (see legend colors) and TDA treatment concentrations are indicated.

**Figure 6:**
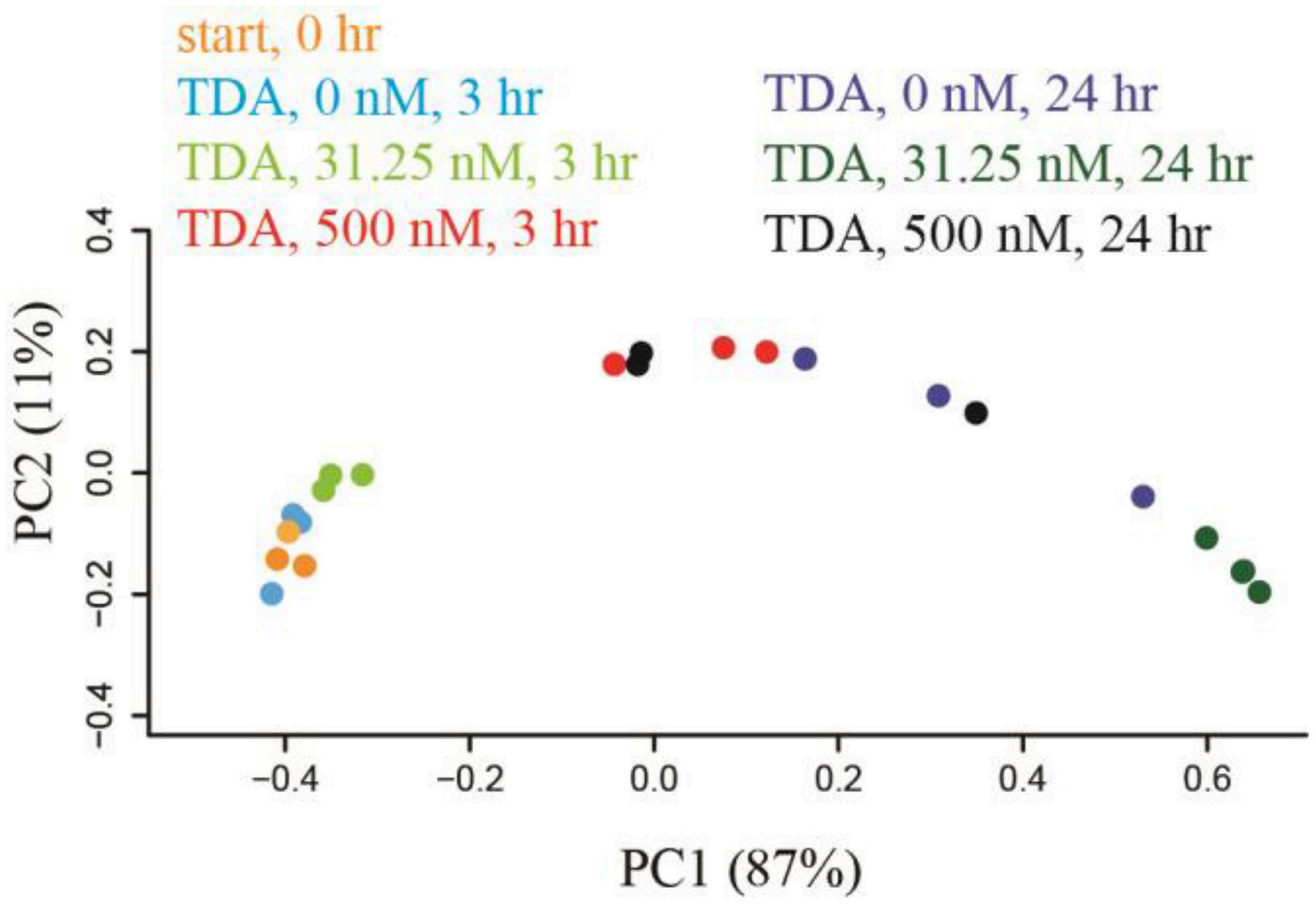
**Microbiota were shifted by TDA addition in a dose-dependent manner within 3 h treatment based on weight-Unifrac PCoA with respect to treatment time and TDA concentration**. TDA (500 nM)-treated microbiota shifted the microbiota structure away from 3 h-microbiota cluster toward the 24 h-group.

Correlation network analysis led us to hypothesize that bacterial species from *Rhodobacteraceae* are key species in structuring the microbial community associated with *N. salina*. To gather evidence for this hypothesis, we treated *N. salina* containing microbial communities with varying concentrations of TDA, a hallmark antibiotic in many species of the *Roseobacteria* clade in *Rhodobacteraceae*, and sampled the microbiota at 3 and 24 h time periods. Taxonomic distributions at the family-level across TDA-perturbed samples, grouped by exposure time, are illustrated in Figure 5. The largest taxa in all microbiota were populations of *Flavobacteriia, Alphaproteobacteria*, and *Gammaproteobacteria* bacterial classes. Figure 5 also shows that the 24 h microbiota decreased in relative abundance of *Flavobacteriia* (23 ± 4%) and *Alphaproteobacteria* (14 ± 4%), comparted to 3 h (excluding TDA, 500 nM, 3 h) *Flavobacteriia* (39 ± 2%) and *Alphaproteobacteria* (32 ± 2%). Meanwhile, *Gammaproteobacteria* relative abundance at 24 h increased to 60 ± 9% compared to 3 h (excluding TDA, 500 nM, 3 h) *Gammaproteobacteria* (25 ± 9%). Of particular note, the microbiota (TDA, 500 nM, 3 h) was composed of *Flavobacteriia* (31 ± 1%), *Alphaproteobacteria* (21 ± 0%), and *Gammaproteobacteria* (45 ± 2%), suggesting it exhibits some compositional similarities to 24 h microbiota (Figure 5). PCoA of the data revealed patterns of beta-diversity (unweighted UniFrac distance metric) primarily forming two clusters differentiated by the time point at which the microbiota was sampled (Figure 6). Specifically, without TDA treatment, the microbiota at the 3-h time point were similar in structure to the starting microbiota (Unifrac distance, 0.03 ± 0.01). The addition of low doses of TDA (31.25 nM) slightly shifted the microbial composition over the 3-h time period (Unifrac distance, 0.05 ± 0.02). In contrast, treatment with higher TDA doses (500 nM) markedly shifted the composition away from the initial microbiota (Unifrac, 0.14 ± 0.02), and at the 3-h time point clustered with the samples obtained after 24 h. Hence, the data imply that microbiota exposed to 500 nM TDA will not only drastically restructure (as compared to the unperturbed community), but also hasten the progression of microbial community structure in a dose-dependent manner toward the more mature microbiota.

## Discussion

Among the efforts to understand the complexity of microalgae microbiota, this current study is unique in that it aimed to chart multispecies relationships underpinning the substructure of microalgae microbiota. To achieve this aim, we experimentally perturbed a multispecies algal microbiota with various chemical treatments, quantified the changes to taxa profiles, and applied network analysis for modeling of interspecies relationship which provided a cohesive overview of the microbial community associated with *N. salina* structure.

It is well established that antibiotics can cause major disturbances within the ecological balance of microbiomes (Mayali and Azam, 2004). Thus, we postulated that antibiotic treatment provides an inferable means to introduce variations among bacterial species due to differential antibiotic susceptibility among them. Indeed, chemical treatments introduced major variations among the microbial communities within the same treatment time. High doses of antibiotic treatment noticeably shifted the overall microbiota structure. Thus, the generated 16S gene metagenomics profiles generally reflected microbiota changes in response to chemical perturbations.

These observations highlight two strengths of correlation network approach. First, rather than listing pair-wise correlations, all correlations were integrated into a unified network of interacting species, making it possible to identify small substructures within the microbiota. While this model is generated only by first-order correlations among species, it presents rich, albeit indirect, information on microbiota community structure, allowing us to explore and test species interactions and prioritize hypotheses. Specifically, the bacterial OTU variations resulting from a set of time-series chemical treatments allowed us to identify five modules emerging out of the complex *N. salina* microbial community. We demonstrated that members of *Rhodobacteraceae* dominated the brown OTUs module, which was significantly associated with ampicillin treatment (Figure 3). Reports have shown that species of the *Roseobacter* clade encode intrinsic β-lactamases and are resistant to β-lactam antibiotics such as ampicillin, with tolerance up to 500 μg/ml (Peng et al., 2011; Treangen et al., 2013). This is 10-fold greater than our high dose ampicillin treatment of 50 μg/ml. Meanwhile, bacteriostatic tetracycline, which inhibits bacteria from reproducing (Peng et al., 2011), surprisingly did not result in the formation of modules. Lack of module formation from tetracycline treatment in our experiments can be explained by the possibility of tetracycline activities being compromised by the formation of complexes with divalent cations (such as Mg^2+^) prevalent in F/2 medium (Lambs et al., 1988; Treangen et al., 2013). In comparison, general metabolites such as glucose did not perturb the network significantly as evidenced by the absence of modules emerging from glucose-supplemented cultures. Overall, these results reflect that an OTU co-abundance network generated correlated associations from empirical data that recapitulates biological information, and therefore has value for further inference analysis.

While correlation analyses do not offer direct mechanistic interpretations, the formation of modules might stem from, for example, between-bacteria metabolic cross-feedings, biofilm formation of aggregated sub-communities, species couplings via chemical signaling or toxic compounds, which could result in bacterial species-species abundance correlations in 16S gene profile data sets. With our data sets, it appears the correlation network has applicability in mining subsets of microbial lineages that share common responsiveness to acute perturbation such as antibiotic treatment and is perhaps less applicable to those stemming from treatments (such as with ubiquitous metabolites) that do not significantly perturb bacterial abundance. Nevertheless, the integration of correlations into interconnected networks may be applied in bridging from bacterial species' activity to microbial community functionality.

Network centrality analysis has been successfully applied in multiple fields (Jeong et al., 2001; Rho et al., 2010; Duran-Pinedo et al., 2011), and we used it to mine influential bacterial species underpinning the interaction network of the microbial community associated with *N. salina*. The top *Rhodobacteraceae* OTU has low connectivity, suggesting it has few interaction with other OTUs in the network. Meanwhile, it has higher betweenness values compared to other OTUs. This pattern suggests modular organization of the network via a minimal number of connections required for information flow. These high-betweenness, low-connectivity OTUs may be conceptually thought of as “bridges” in the non-redundant shortest paths, the observation of which resembles previously reported properties of other biological networks (Joy et al., 2005). In comparison, the high-connectivity, low-betweenness case of *Alteromonadacea* suggests it lies on a large number of redundant shortest paths between other vertices. The difference between the network inferences of *Rhodobacteraceae* and *Alteromonadacea* is likely linked to distinct biological and ecological roles of these two groups. *Rhodobacteraceae*, which contains the *Roseobacter* clade, are one of the most ubiquitous and abundant bacterial lineages associated with phytoplankton both in environmental and laboratory cultures (Geng and Belas, 2010b; Amin et al., 2012) and has been shown to form close relationships with microalgae (Treangen et al., 2013; Kelder et al., 2014). The interactions among members of the *Roseobacter* clade (*Alphaproteobacteria*) and microalgae may occur through physical attachment (Miller and Belas, 2006; Mayali et al., 2008; Krohn-Molt et al., 2013), exchange of nutrients and metabolites (Keshtacher-Liebso et al., 1995; Howard et al., 2006), or antibiotics and signaling molecules (Brinkhoff et al., 2004; Seyedsayamdost et al., 2011; Amin et al., 2015). On the other hand, the marine bacteria from *Alteromonadaceae* are dominant phylotypes among microbial communities in response to the flux of organic matter in phytoplankton blooms, demonstrating how species of *Alteromonadaceae* could participate in structuring microalgae bacterial communities (McCarren et al., 2010; Tada et al., 2011).

To explore the possibility of the key species having influential effects on the overall configuration of the microbiota, we looked for evidence of microbiota responsiveness to the proposed key species. We subjected the *N. salina* microbial communities to varying doses of TDA, which is secreted as a hallmark bioactive compound in many species of the *Roseobacter* clade from *Rhodobacteraceae* family (Berger et al., 2011). TDA has both antibiotic and transcription induction activity in a subgroup of algae-associated bacterial genera that include *Phaeobacter, Silicibacter, Ruegeria*, and *Pseudovibrio* (Berger et al., 2011). Interestingly, we found that in general the relative abundance of *Rhodobacteraceae* decreased, while bacteria in *Alteromonadales* order of unclassified family increased in composition (Figure 5). These observed patterns between TDA and taxa composition are less clear since TDA activity to *Alteromonadales* has not been documented. Meanwhile the large total number of bacterial populations and differential bacterial growth rates in microbiota might have compounded the observed relative abundance. In terms of community structure, we observed shifting of microalgae microbiota induced by TDA dosage, and a 3 h 500 nM TDA treatment sufficiently skewed the original microbiota structure toward the 24 h unperturbed microbiota. This suggests that, given the myriad complexity in microbiota, microbial community associated with *N. salina* potentially harbors a dynamic property that responds to the presence of infochemicals exemplified by TDA. While the concentration of TDA produced naturally in either sea water or within the phycosphere is not documented, the involvement of bioactive compounds such as TDA in modulating microbiota structure has profound implications for bacterial community assemblages. Indeed, multiple bioactive compounds with various activities and specificities, such as indole-3-acetic acid (Amin et al., 2015) and indigoidine (Cude et al., 2012), have been characterized in many *Roseobacter* strains from microalgae microbiota (Buchan et al., 2005; Cude et al., 2012; Leiman et al., 2013; Treangen et al., 2013). In this context, the chemical composition and quantities of bioactive compounds from key species in microbiota may play a role in fine tuning of microbiota to different microbiota compositions and structures.

Our finding from network analysis therefore offers a tangible experimental metagenomics framework to tackle the following question: what is the structure of microbial community associated with *N. salina* if modeled from the species-species correlation network? Module formation in the microbiota network helps bridge the gap between deep knowledge of individual keystone OTUs and a systems-level view of the microbial community. But such application will have to tackle several issues. Given the connectivity of the correlation network, the correlation network model does not provide detailed mechanisms nor offer regulatory ramification of such variations. While accurate inter-OTU correlation analysis remains an area of active research, methods developed recently such as CoNet (Faust et al., 2012), SparCC (Friedman and Alm, 2012), SPIEC-EASI (Kurtz et al., 2015), and MENA (Deng et al., 2012) could potentially be used to analyze inter-OTUs relationships in microalgae microbiota to improve association accuracy. Meanwhile, many of the remaining OTUs in modules have limited functional annotations due to the nature of 16S amplicon method. Such limitation, however, could be solved using metagenomics combined with metatranscriptomics to better understand microbiota roles in the ecosystem. In the long term, delineation of the substructure of the microbial community associated with *N. salina* may help us detect, investigate, and assess practical responsiveness of “key” species or probiotic species introduced into the microbiome to withstand environmental perturbations and improve microalgae production.

## Author contributions

KS, TL, EY designed experiments. HG, EY, MT performed experiments. HG, KS analyzed and modeled data. HG, TD, EY, KS wrote manuscript.

### Conflict of interest statement

The authors declare that the research was conducted in the absence of any commercial or financial relationships that could be construed as a potential conflict of interest.

## Supplementary material

Figure S1 **Modules of OTUs in chemical treated microbiotas network**. Hierarchical clustering dendrogram of OTUs was built based on dissimilarity based on nodes topological overlap. Modules have been colored by module membership.
