# Supplementary figures and images for: Changes in the Structure of the Microbial Community Associated with Nannochloropsis salina following Treatments with Antibiotics and Bioactive Compounds
Source: Front Microbiol. 2016 Jul 26;7:1155. doi: 10.3389/fmicb.2016.01155 (PMC4960269; doi:10.3389/fmicb.2016.01155)

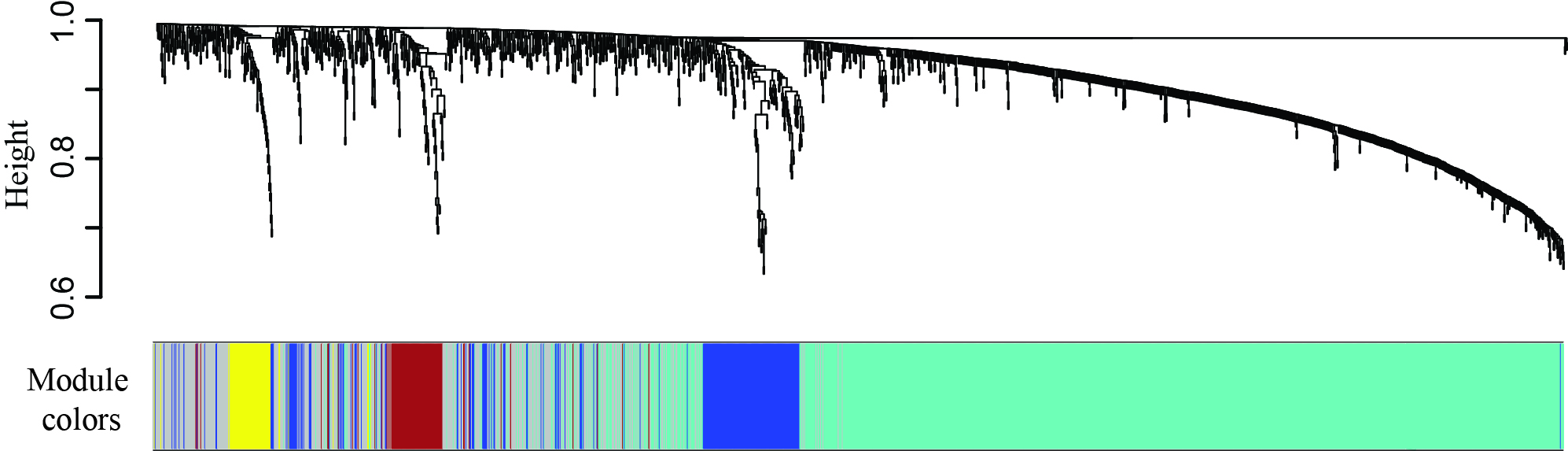

Supplement: Figure S1 — Modules of OTUs in chemical treated microbiotas network. Hierarchical clustering dendrogram of OTUs was built based on dissimilarity based on nodes topological overlap. Modules have been colored by module membership. [file Image1.JPEG]
